# Higher anticoagulation targets and risk of thrombotic events in severe COVID-19 patients: bi-center cohort study

**DOI:** 10.1186/s13613-021-00809-5

**Published:** 2021-01-25

**Authors:** Julie Helms, François Severac, Hamid Merdji, Maleka Schenck, Raphaël Clere-Jehl, Mathieu Baldacini, Mickaël Ohana, Lélia Grunebaum, Vincent Castelain, Eduardo Anglés-Cano, Laurent Sattler, Ferhat Meziani, for the CRICS TRIGGERSEP Group (Clinical Research in Intensive Care Sepsis Trial Group for Global EvaluationResearch in Sepsis)

**Affiliations:** 1 Service de Médecine Intensive Réanimation, Nouvel Hôpital Civil, Hôpitaux Universitaires de Strasbourg, 1, Place de l’Hôpital, 67091 Strasbourg Cedex, France; 2 ImmunoRhumatologie Moléculaire, INSERM UMR_S1109, LabEx TRANSPLANTEX, Centre de Recherche d’Immunologie et d’Hématologie, Faculté de Médecine, Fédération Hospitalo-Universitaire (FHU) OMICARE, Fédération de Médecine Translationnelle de Strasbourg (FMTS), Université de Strasbourg (UNISTRA), Strasbourg, France; 3 Groupe Méthodes en Recherche Clinique (GMRC), Hôpital Civil, Hôpitaux Universitaires de Strasbourg, Strasbourg, France; 4 INSERM (French National Institute of Health and Medical Research), UMR 1260, Regenerative Nanomedicine (RNM), FMTS, Strasbourg, France; 5 Service de Médecine Intensive Réanimation, Hôpitaux Universitaires de Strasbourg, Hautepierre, Strasbourg, France; 6 Radiology Department, Nouvel Hôpital Civil, Strasbourg University Hospital, Strasbourg, France; 7 Laboratoire d’Hématologie, Hôpitaux Universitaires de Strasbourg, Hautepierre, Strasbourg, France; 8 Innovative Therapies in Haemostasis, INSERM UMR_S 1140, Université de Paris, 75006 Paris, France

**Keywords:** Anticoagulation, Coagulopathy, COVID-19, Pulmonary embolism, SARS-CoV-2, Thrombosis

## Abstract

**Background:**

Thromboprophylaxis of COVID-19 patients is a highly debated issue. We aimed to compare the occurrence of thrombotic/ischemic events in COVID-19 patients with acute respiratory distress syndrome (ARDS) treated with either prophylactic or therapeutic dosage of heparin. All patients referred for COVID-19 ARDS in two intensive care units (ICUs) from two centers of a French tertiary hospital were included in our cohort study. Patients were compared according to their anticoagulant treatment to evaluate the risk/benefit of prophylactic anticoagulation versus therapeutic anticoagulation. Medical history, symptoms, biological data and imaging were prospectively collected.

**Results:**

One hundred and seventy-nine patients (73% men) were analyzed: 108 in prophylactic group and 71 in therapeutic group. Median age and SAPS II were 62 [IQR 51; 70] years and 47 [IQR 37; 63] points. ICU mortality rate was 17.3%. Fifty-seven patients developed clinically relevant thrombotic complications during their ICU stay, less frequently in therapeutic group (adjusted OR 0.38 [0.14–0.94], p = 0.04). The occurrences of pulmonary embolism (PE), deep vein thrombosis (DVT) and ischemic stroke were significantly lower in the therapeutic group (respective adjusted OR for PE: 0.19 [0.03–0.81]; DVT: 0.13 [0.01–0.89], stroke: 0.06 [0–0.68], all p < 0.05). The occurrence of bleeding complications was not significantly different between groups, neither were ICU length of stay or mortality rate. D-dimer levels were significantly lower during ICU stay, and aPTT ratio was more prolonged in the therapeutic group (p < 0.05).

**Conclusion:**

Increasing the anticoagulation of severe COVID-19 patients to a therapeutic level might decrease thrombotic complications without increasing their bleeding risk.

## Background

The severe acute respiratory syndrome coronavirus-2 (SARS-CoV-2) is responsible for an intense systemic inflammatory syndrome and an endotheliopathy leading to coagulation activation [1–4]. COVID-19 patients therefore have a procoagulant state that may lead to thrombotic complications despite pharmacological thromboprophylaxis. In a cohort of patients admitted to the intensive care unit (ICU) for hypoxemic acute respiratory failure due to COVID-19, we have shown a high occurrence of pulmonary embolisms (16.7%), usually diagnosed a few days after ICU admission because of worsening of hypoxemia [5]. The occurrence of pulmonary embolism was higher in COVID-19 acute respiratory distress syndrome (ARDS) than in non-COVID-19 ARDS (11.7 versus 2.1%) despite prophylactic anticoagulation.

Interestingly, pulmonary embolism was not necessarily associated to deep venous thrombosis [6], raising the hypothesis of a thrombotic rather than a thrombo-embolic mechanism. The intense inflammation might indeed affect the alveolar vascular endothelium from the early stages of the disease, leading to a lung-specific origin of coagulopathy and resulting in the formation of in situ pulmonary clots [3, 6–8]. Besides this local coagulation activation, the endotheliopathy might also play a major role in COVID-19-associated coagulopathy pathogenesis [9].

Despite the multiplication of recent reports on thrombotic complications in COVID-19 critically ill patients [5, 10–12], it has been argued that current data do not support the use of full intensity anticoagulation doses unless otherwise clinically indicated.

Our hypothesis is, however, that therapeutic anticoagulation might decrease the occurrence of life-threatening thrombotic complications in patients with severe forms of COVID-19. We have therefore compared the occurrence of any thrombotic/ischemic event in COVID-19 ARDS patients admitted to ICU and treated with either prophylactic (prophylactic group) or therapeutic dosage of heparin (therapeutic group).

## Patients and methods

### Design

The present study was a before- (prophylactic)/after- (therapeutic) one: before the evidence of high risk of thrombotic/ischemic events in COVID-19 patients, COVID-19 patients received prophylactic anticoagulation, except if they had an indication for therapeutic anticoagulation; after the publication of the French recommendations in April 2020 (French version) recommending intensification of anticoagulation in COVID-19 patients are high risk of thrombotic/ischemic events, including ICU patients, the two ICUs modified their anticoagulation protocol according to these recommendations [13].

### Patients

Between March 3rd and May 30th 2020, all patients referred for ARDS [14] due to SARS‐CoV‐2 were included on admission to two ICUs in two centers of a French tertiary hospital. Patients were managed following current guidelines without specific therapeutic intervention [15]. Approval was obtained from the local ethics committee of the University Hospital of Strasbourg (reference CE-2020-34). All demographic characteristics, medical history, clinical signs, biological and imaging data were prospectively collected. Data were analyzed on August the 10th (i.e., 80 days of follow-up for the most recently admitted patients).

“Prophylactic group” included patients treated with standard or reinforced prophylactic dosage of heparin (low molecular weight heparin LMWH-enoxaparin—up to 6000 IU/12 h SC in obese patients or unfractionated heparin UFH 200 IU/kg/24 h if creatinine clearance < 30 mL/min). Therapeutic anticoagulation included patients who received LMWH at curative dose (100 IU/kg/12 h SC based on actual weight, without exceeding 10,000 IU/12 h or UFH 500 IU/kg/24 h if creatinine clearance < 30 mL/min) according to the French recommendations [13]. Monitoring of anticoagulants was performed according to recommendations [13].

If a patient from the prophylactic group developed a thrombotic/ischemic complication requiring therapeutic dosing of heparin during ICU stay, she/he received the appropriate therapeutic dosing of heparin as soon as the thrombotic/ischemic complication was diagnosed, but she/he was analyzed in the “prophylactic group”.

**Fig. 1:**
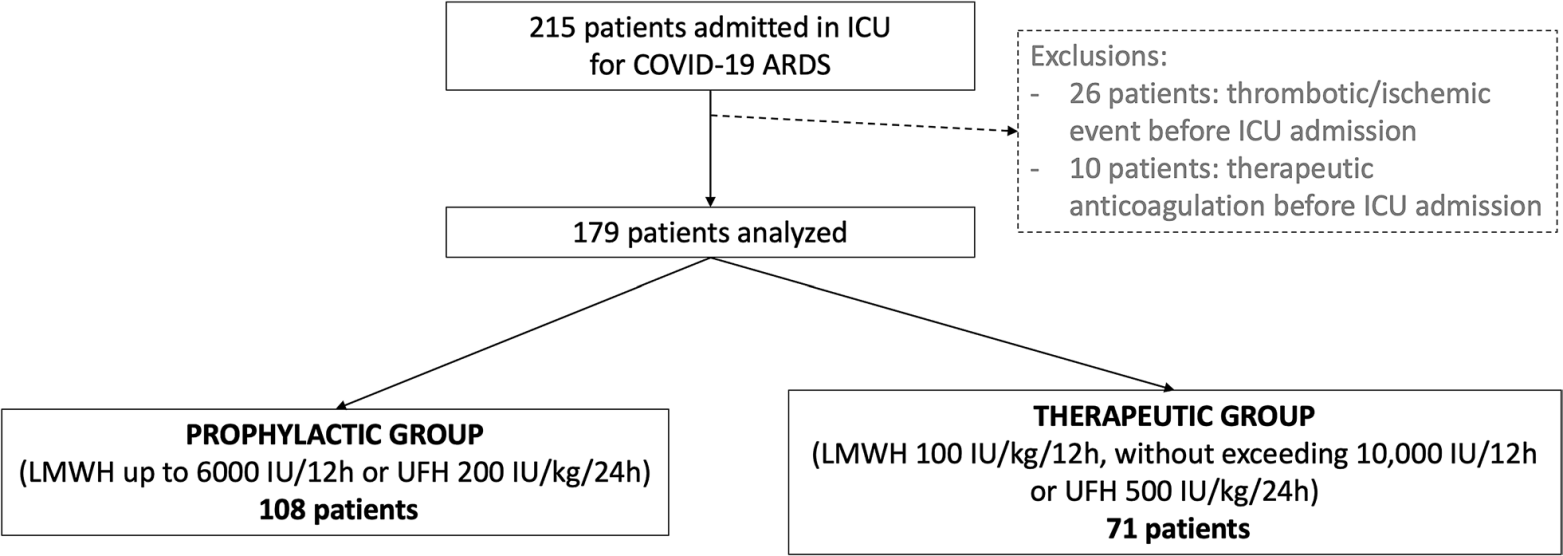
Flow chart

Patients were excluded if: (i) they were diagnosed with a thrombotic/ischemic event before or on the day of ICU admission; (ii) they had any contra-indication to anticoagulation, (iii) they were already under therapeutic anticoagulation on ICU admission (Fig. 1, flowchart).

Patients under prophylactic anticoagulation at ICU admission were eligible if they had no exclusion criteria.

Two hundred and fifteen consecutive patients, with positive real-time reverse transcriptase PCR tests for COVID-19, admitted to one of the ICUs for ARDS were included in the study. Twenty-six patients diagnosed with a thrombotic event before ICU admission and 10 patients under therapeutic anticoagulation were excluded (Fig. 1, flowchart). No patient was excluded because of contra-indication to anticoagulation.

### Outcomes

The primary end-point was to compare the occurrence of any thrombotic/ischemic event in patients admitted in ICU for COVID-19 ARDS in prophylactic and therapeutic groups.

The secondary objectives were: (i) To compare the occurrence of each type of thrombotic/ischemic events occurring during ICU stay: deep vein thrombosis, pulmonary embolism, ischemic stroke, limb/extremity ischemia, myocardial infarction, cerebral stroke, ECMO circuit thrombosis, renal replacement therapy (RRT) device thrombosis; (ii) To compare the occurrence of hemorrhagic events requiring transfusion (grade 3 of World Health Organization bleeding scale) or life-threatening bleeding complication during ICU stay defined by (grade 4 of World Health Organization bleeding scale); (iii) To compare the ICU length of stay and mortality rates; (iv) To compare the effect of anticoagulant treatment on hemostasis in both groups during ICU stay.

### Laboratory analysis

Platelet count and coagulation tests were performed daily during the ICU stay, including prothrombin time (PT), antithrombin activity (AT), fibrinogen, D-dimers and activated partial thromboplastin time (aPTT). Factor V (FV), von Willebrand factor (vWF) antigen, vWF activity, and factor VIII (FVIII) activity were performed. Lupus anticoagulant was searched when a coagulation disorder was suspected, based on a prolonged aPTT at ICU admission or on the occurrence of a thrombotic event during ICU stay. Please refer to online supplemental material for further details.

### Imaging

CT angiography (pulmonary/abdomino-pelvic/lower limbs) was performed during ICU stay according to clinical or laboratory parameters evolution suggesting thrombosis, as previously described [5]. In particular, according to the following predefined protocol, pulmonary embolism was suspected if PaO_2_/FiO_2_ worsened despite inhaled nitric oxide/prone positioning, if hemodynamic was impaired requiring fluid challenge and/or increased norepinephrine infusion rate, evidence of dilated right ventricle—even without acute cor pulmonale, rapid elevation of D-dimer despite anticoagulation.

Patients with suspicion of stroke, based on pathological neurological examination, had either a non-contrast brain CT and/or a brain magnetic resonance imaging (MRI) with diffusion weighted imaging and 3D FLAIR acquisitions.

All CT and MR examinations were read by consultant radiologists specialized in emergency radiology.

### Statistics

Continuous variables are presented as median with the first and third quartile and were compared using non parametric Wilcoxon tests. Categorical variables are presented as counts and proportions and were compared using Pearson’s χ^2^ tests or Fisher’s exact tests. The occurrence of life-threatening thrombotic complications was compared between the groups (prophylactic versus therapeutic) using multivariable logistic regression models. The comparisons were adjusted on the baseline characteristics that were unbalanced between groups or had clinical relevance (age at admission, sex, history of venous thrombo-embolic event, diabetes, chronic renal diseases, PaO_2_/FiO_2_ ratio, antiviral treatment, renal replacement therapy, coagulation parameters at admission as d-dimer level, AT, prothrombin time and factor V and UFH). For the comparison of strokes, we used Firth’s bias reduction method to deal with the problem of separation in logistic regression [16, 17]. Results are presented as odds ratio with 95% confidence intervals. Sensitivity analyses were performed using a propensity score. The score was created using a logistic regression model with the treatment variable as the independent variable and the set of adjustment variables from the multivariable model as the dependent variables. The treatment effect was then estimated by adjusting on the propensity score. A p-value < 0.05 was considered as statistically significant. All the analyses were performed using R software version 3.6.1. R Core Team (2019). R: a language and environment for statistical computing. R Foundation for Statistical Computing, Vienna, Austria. URL https://www.R-project.org/.

## Results

### Characteristics of the patients

Among the 179 analyzed patients (Fig. 1), median age was 62 [51; 70] years old and included 130 men (72.6%). The median of simplified acute physiology score (SAPS) II was 47 [37; 63] points and median PaO_2_/FiO_2_ was 123 [95; 168] on admission. The median length of stay in ICU was 8 [4; 13] days; all the patients were discharged from ICU at the time of data analysis. Mortality rate was 17.3% (31 patients).

Patient characteristics of both prophylactic and therapeutic groups are summarized in Table 1.

**Table 1:** Characteristics of COVID-19 ARDS

	All patients (n = 179)	Prophylactic Group (n = 108)	Therapeutic Group (n = 71)	p
Age—median, IQR	62 [51; 70]	61 [51; 70]	64 [53; 71]	0.56
Male—n (%)	130 (72.6)	83 (76.9)	47 (66.2)	0.12
BMI (kg/m^2^)—median, IQR	30 [26; 34]	29 [26; 33]	31 [27; 34]	0.25
Medical history—n (%)				
Malignancies/hemopathies	8 (4.5)	4 (3.7)	4 (5.6)	0.79
Cardiovascular diseases	76 (42.4)	43 (39.8)	33 (46.5)	0.38
Thrombo-embolic event	9 (5.0)	7 (6.5)	2 (2.8)	0.46
Cerebrovascular diseases	7 (3.9)	3 (2.8)	4 (5.6)	0.56
Immune diseases	2 (1.1)	2 (1.9)	0 (0.0)	0.73
Diabetes	31 (17.3)	13 (12.0)	18 (25.3)	0.02
Chronic liver disease	3 (1.7)	2 (1.9)	1 (1.4)	1
Chronic renal disease	16 (8.9)	5 (4.6)	11 (15.5)	0.02
Respiratory disease	20 (11.2)	12 (11.1)	8 (11.3)	0.97
Baseline severity scores				
SAPS II—median, IQR	47 [37; 63]	48 [37; 63]	47 [38; 62]	0.72
SOFA—median, IQR	8 [5; 10]	8 [5; 10]	8 [5; 10]	0.90
Anticoagulation treatment in ICU				
LMWH—n (%)	115 (64.2)	87 (80.6)	28 (39.4)	< 0.05
UFH—n (%)	64 (35.8)	21 (19.4)	43 (60.6)	< 0.05
Supportive treatments				
Invasive mechanical ventilation—n (%)	179 (100)	108 (100)	71 (100)	1
RRT—n (%)	35 (19.6)	16 (14.8)	19 (26.8)	0.05
ECMO—n (%)	11 (6.2)	5 (4.6)	6 (8.5)	0.47
ECMO duration (days)—median, IQR	7.0 [6.5; 11.0]	7.0 [7.0; 11.0]	8.0 [6.3; 10.5]	1
Outcome				
ICU length of stay (days)—median, IQR	10 [5; 19]	9 [5; 18]	10 [6; 19]	0.27
ICU mortality—n (%)	31 (17.3)	20 (18.5)	11 (15.5)	0.67

*ARDS* acute respiratory distress syndrome, *BMI* body mass index, *ECMO* extracorporeal membrane oxygenation, *ICU* intensive care unit, *IQR* interquartile range, *LMWH* low molecular weight heparin, *RRT* renal replacement therapy, *SOFA* sequential organ failure assessment, *SAPSII* simplified acute physiology score II, *UFH* unfractionated heparin

**Fig. 2:**
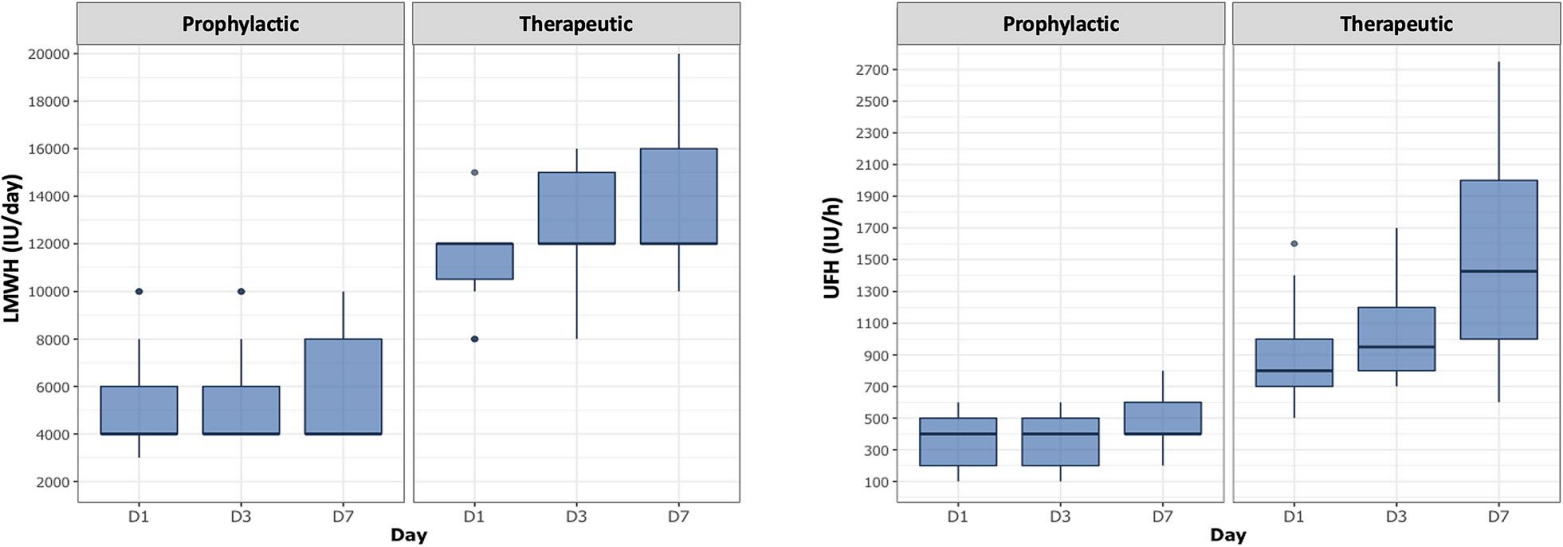
Anticoagulation dosage in therapeutic and prophylactic groups at days 1, 3 and 7 after ICU admission. *IU* international unit, *LMWH* low molecular weight heparin, *UFH* unfractionated heparin

One hundred and eight patients were included in prophylactic group and 71 in therapeutic group (Fig. 1). Heparin doses were significantly higher in therapeutic group than in prophylactic group during ICU stay (p < 0.05 for all comparisons) (Fig. 2).

### Rates of thrombotic/ischemic complications and severe bleeding events

Fifty-seven patients (31.8%) developed at least one clinically relevant thrombotic event during their ICU stay, which were less frequent in the therapeutic group (adjusted OR at 0.38 [0.14–0.94], p = 0.04) (Table 2).

**Table 2:** Thrombotic and ischemic complications depending on anticoagulant treatment

	All patients (n = 179)	Prophylactic group (n = 108)	Therapeutic group (n = 71)	Univariable analysis		Multivariable analysis	
				OR [95% CI]	p	OR [95% CI]	p
Thrombo-embolic complications—n (%)	57 (31.8)	42 (38.9)	15 (21.1)	0.42 [0.20–0.88]	0.01	0.38 [0.14–0.94]	0.04
Pulmonary embolisms – n (%)	25 (14.0)	22 (20.4)	3 (4.2)	0.17 [0.03–0.61]	< 0.01	0.19 [0.03–0.81]	0.04
Deep vein thrombosis—n (%)	11 (6.1)	10 (9.3)	1 (1.4)	0.13 [0–1.39]	0.18	0.13 [0.01–0.89]	0.04
Cerebral ischemic attack—n (%)	6 (3.4)	6 (5.6)	0	0 [0–1.27]	0.09	0.06 [0–0.68]	0.02
RRT filter/thrombosis—n (%)	32 (17.9)	19 (17.6)	13 (16.7)	0.97 [0;41–2.24]	0.94	1.04 [0.34–3.11]	0.95
Nb of RRT filter per day of RRT—median, IQR	3.0 [1.0; 6.8]	3.0 [2.0; 7.0]	2.0 [1.0; 5.0]	/	0.52	/	/

*CI* confidence interval, *IQR* interquartile range, *OR* odds ratio, *RRT* renal replacement therapy

One hundred eleven CTPA were performed to investigate the cause of a respiratory reaggravation or because of a significant increase in d-dimers, and allowed the diagnosis of 25 troncular, lobar or segmental pulmonary embolisms (14.0%) (24 men—96%, median age 60 [48; 70] years old). PE was diagnosed at 6.5 [4.0; 13.3] days (median, IQR) after ICU admission. Multivariable analysis showed that the occurrences of PE and deep vein thrombosis were significantly higher in the prophylactic group (20.4% and 9.3%, respectively) than in the therapeutic group (4.2% and 1.4%, respectively), with respective adjusted odds ratios at 0.19 [0.03–0.81] and 0.13 [0.01–0.89], p < 0.05 for both comparisons. The results remained unchanged in the sensitivity analyses (Additional file 1: Table 1).

Six ischemic strokes were diagnosed on brain CT or MRI. The occurrence of ischemic stroke was 5.6% (6 patients, 4 men—67%, median age 60 [58; 65] years) in the prophylactic group, versus 0% in the therapeutic group, with an adjusted OR 0.06 [0–0.68], p = 0.02.

Occurrences of circuit clotting of continuous RRT (on dialysis catheters, with similar anticoagulation protocols of RRT in all patients), median lifespan of an RRT circuit, thrombotic occlusions of ECMO centrifugal pump, acute limb ischemia were not significantly different between groups. No patients suffered from myocardial infarction, mesenteric ischemia, digital/toes necrosis or purpura during their ICU stay.

The occurrence of severe bleeding complications was not significantly different between the two groups, with 2 bleeding complications in the prophylactic group (1 hemorrhage on ECMO canulae and 1 gastro-intestinal bleeding) and 1 in therapeutic group (1 gastro-intestinal bleeding).

### Therapeutic anticoagulation failed to improve prognosis of critically ill ARDS patients with COVID-19

ICU length of stay and mortality rate did not differ between groups (Table 1).

### Coagulation parameters

**Fig. 3:**
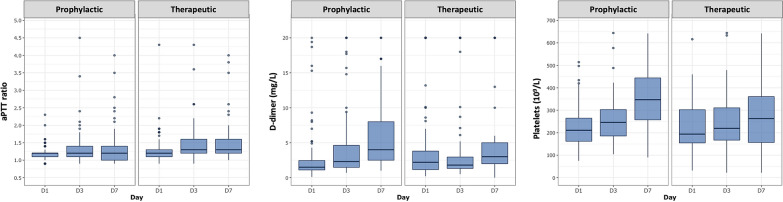
Kinetics of coagulation parameters in therapeutic and prophylactic groups at days 1, 3 and 7 after ICU admission

Consistent with higher anticoagulation levels, aPTT ratio was significantly more prolonged in the therapeutic group versus prophylactic group at days 3 and 7 (p < 0.05) (Fig. 3). d-dimer levels were significantly lower in the therapeutic group versus the prophylactic group (p < 0.05) at days 3 and 7, and platelets, although within normal ranges in all patients, were significantly decreased at day 7 in the therapeutic group versus prophylactic group (p < 0.05) (Fig. 3). Factor VIII was also significantly decreased in the therapeutic group versus the prophylactic group (254 [199; 285] versus 345 [265; 428], p < 0.05). In the therapeutic group, median aPTT ratio was 1.4 [1.2; 1.7] and median anti-Xa 0.30 [0.18; 0.43] IU/mL during ICU stay. Other routine coagulation parameters (prothrombin time, factor V, antithrombin and fibrinogen) did not significantly differ at days 3 and 7 (data not shown).

Seventy-six out of the 93 patients tested (81.7%) had positive lupus circulating anticoagulant during their ICU stay (40/50 patients—80.0% in prophylactic group and 36/43 patients—83.7% in therapeutic group, NS). Median levels of von Willebrand factor are provided in Additional file 1: Table 1, but did not significantly differ between groups.

## Discussion

Our team has recently shown that COVID-19 ARDS was associated with an increased risk of thrombotic events compared to other ARDS etiologies despite prophylactic anticoagulation [5]. In the absence of randomized trials, there was an urgent need to evaluate real-world evidence related to outcomes with the use of therapeutic anticoagulation versus standard prophylactic anticoagulation in COVID-19 patients with ARDS. We therefore proposed to consider higher anticoagulation targets than in usual critically ill patients. Our observational bi-center cohort suggests that higher anticoagulation targets for thromboprophylaxis of severe COVID-19 patients could decrease the rate of thrombosis, without increase in severe bleeding events and without difference in ICU mortality or length of stay compared with patients in regular prophylaxis.

Despite recent evidence that critically ill COVID-19 patients are at high risk of thrombotic complications [5, 10–12], most experts guidelines recommended prophylactic levels of anticoagulation [18, 19]. The French Working Group on Perioperative Hemostasis (GIHP) and the French Study Group on Thrombosis and Hemostasis (GFHT), however, proposed that heparin treatment should be intensified in the context of COVID-19 on the basis of clinical and biological criteria of severity, especially in severely ill ventilated patients, for whom the diagnosis of pulmonary embolism cannot be easily confirmed [13].

Curative anticoagulant treatment of COVID-19 patients might limit COVID-19 associated coagulopathy, but also decrease endothelial dysfunction, as assessed by significantly lower level of circulating endothelial cells in patients treated with curative anticoagulation prior to admission [20].

Consistent with previous data [5, 21, 22], we have also showed that bleeding events are uncommon in COVID-19 patients despite therapeutic anticoagulation, and their occurrence was not increased by higher anticoagulation targets in our study. Al-Samkari et al. [22] indeed reported a 2.3%-rate of major bleeding complications (WHO grade 3–4). Considering the high rate of thrombotic events, risk benefit balance would therefore favor higher anticoagulation targets. Empirical higher anticoagulation targets were also used in ARDS patients during 2009 swine flu pandemic, which reduced the incidence of thrombotic event without increasing bleeding complications [23].

Interestingly, despite standard therapeutic doses of anticoagulation, we only reached “mild” anticoagulation targets (median aPTT ratio 1.4 [1.2; 1.7] and median anti-Xa 0.30 [0.18; 0.43] IU/mL), emphasizing the difficulties to anticoagulate COVID-19 patients due to procoagulant feature and heparin resistance [1, 24]. However, d-dimer levels were significantly lower in the therapeutic group, suggesting that fibrinolysis (and therefore thrombosis) was decreased in these patients.

Recommending higher levels of anticoagulation in the most severe patients may thus be a cornerstone of the thromboprophylaxis in COVID-19 patients. However, it is likely that this strategy will not be sufficient to completely prevent thrombotic events. Indeed, pulmonary embolism was most frequently diagnosed a few days after ICU admission, although a significant number was diagnosed before or by the time of ICU admission (12.1%) and were therefore excluded from the present study. The thrombotic process probably begins in the early inflammatory course of COVID-19, even whilst patients are still ambulant at home or on medicine wards, and the risk of thrombotic event is increasing with the progression of the underlying disease [5]. Although Tang et al. [25] suggested that anticoagulant therapy mainly with low molecular weight heparin would be associated with better prognosis in severe COVID-19 patients fulfilling sepsis-induced coagulopathy criteria or with markedly elevated d-dimer, higher anticoagulation targets was not associated with better prognosis in our cohort. Indeed, neither ICU length of stay nor ICU mortality were decreased in therapeutic group compared to prophylactic group.

The current study has several limitations: being an observational study, it can therefore not fully account for unmeasured confounding factors, including non-systematic screening for thrombotic complications for example, and we will therefore not be able to draw reliable conclusions, without further studies. Then, we have included a relatively small number of patients, which may alter the generalizability of the study. Finally, the lack of routine screening for thrombotic events is also a limitation, and has probably led us to under-estimate thrombotic complications. Results from prospective randomized controlled trial will therefore be necessary to confirm our results. Nearly 30 clinical trials comparing different anticoagulation regimens or molecules are currently recruiting or about to start recruitment (Clinicaltrials.gov).

## Conclusion

Our findings highlight the urgent need for randomized control trials comparing anticoagulation targets in COVID-19 patients admitted in ICU. Indeed, our observational study suggests that higher anticoagulation targets for thromboprophylaxis of severe COVID-19 patients could decrease the rate of thrombosis. Higher anticoagulation targets were not associated with increased bleeding and were not associated with a difference in ICU mortality or length of stay compared with patients in regular prophylaxis.

## Supplementary Information

**Additional file 1**: **Table 1** Sensitivity analysis (multivariable model adjusted on the propensity score).

## Data Availability

All data generated or analysed during this study are included in this published article [and its Additional files].

## Abbreviations

aPTT: Activated partial thromboplastin time
ARDS: Acute respiratory distress syndrome
ECMO: Extracorporeal membrane oxygenation
F: Factor
GFHT: French Study Group on Thrombosis and Hemostasis
GIHP: Group on Perioperative Hemostasis
ICU: Intensive care unit
LMWH: Low molecular weight heparin
MRI: Magnetic resonance imaging
OR: Odds ratio
IQR: Interquartile range
RRT: Renal replacement therapy
SAPS: Simplified acute physiology score
SARS-CoV-2: Severe acute respiratory syndrome coronavirus-2
UFH: Unfractionated heparin
vWF: Von Willebrand factor
