# Supplementary material for: Higher anticoagulation targets and risk of thrombotic events in severe COVID-19 patients: bi-center cohort study
Source: Ann Intensive Care. 2021 Jan 25;11:14. doi: 10.1186/s13613-021-00809-5 (PMC7829649; doi:10.1186/s13613-021-00809-5)
Supplement: Supplementary file 1 — Additional file 1: Table 1 Sensitivity analysis (multivariable model adjusted on the propensity score). [file 13613_2021_809_MOESM1_ESM.docx]

**Additional file 1 Table 1: Sensitivity analysis (multivariable model adjusted on the propensity score)**

|  | OR [95%IC] | p |
| --- | --- | --- |
| Thrombosis | 0.41  [0.18 ; 0.88] | 0.025 |
| Pulmonary embolism | 0.23 [0.05 ; 0.79] | 0.033 |
| Deep vein thrombosis | 0.08 [0.01 ; 0.58] | 0.032 |
| Stroke | 0.06 [0.00 ; 0.67 | 0.019 |
| RRT filter thrombosis | 1.02 [0.40 ; 2.53] | 0.974 |

CI: confidence interval; OR: odds ratio; RRT: renal replacement therapy
